# The Evolution of Value-Based Care: Why Cost-Effectiveness Matters in Gastroenterology

**DOI:** 10.14309/crj.0000000000001867

**Published:** 2025-10-15

**Authors:** Sneh Sonaiya, Hareesha Rishabh Bharadwaj, Dushyant Singh Dahiya

**Affiliations:** 1 Department of Internal Medicine, University of Nevada Las Vegas, Las Vegas, NV; 2 Faculty of Biology Medicine and Health, The University of Manchester, Manchester, UK; 3 Division of Gastroenterology, Hepatology and Motility, The University of Kansas School of Medicine, Kansas City, KS

**Keywords:** gastroenterology, healthcare, cost effectiveness, analysis

## INTRODUCTION

Healthcare expenditures in the United States continue to rise, reaching an estimated $4.9 trillion—or $14,570 per capita—in 2023.^1^ Gastroenterology (GI) represents a significant share of this spending. In 2021 alone, GI-related healthcare costs totaled $111.8 billion, and in 2022, an estimated 23.5 million GI endoscopic procedures were performed nationwide.^2^ In this context, the shift toward value-based care underscores the need for gastroenterologists to balance clinical excellence with responsible resource utilization. Cost-effectiveness analysis (CEA) provides a structured framework to guide these choices. When deciding between endoscopic mucosal resection and endoscopic submucosal dissection, considering prophylactic clipping after polypectomy, or determining surveillance intervals, each decision carries not only clinical implications but also economic consequences. By integrating cost-effectiveness into everyday practice, gastroenterologists can align high-quality patient care with healthcare sustainability ensuring that clinical outcomes and resource stewardship move forward hand in hand.^3^

## WHY COST-EFFECTIVENESS MATTERS IN GI

While randomized controlled trials and meta-analyses provide robust data on clinical outcomes, they frequently overlook the economic implications of competing interventions. As a result, cost-effectiveness evidence often trails behind the clinical literature. Importantly, these gaps increasingly intersect with reimbursement models as payors and health systems. For instance, as Gajula et al showed, endoscopic submucosal dissection remained consistently under-reimbursed across insurance types when compared with surgical alternatives, despite its proven clinical efficacy and safety in appropriate patient populations.^4^ Delays in appropriate reimbursement structures can potentially limit the widespread adoption of such interventions. Similarly, Ladabaum et al demonstrated that lowering the starting age of colorectal cancer screening from 50 to 45 years was cost-effective, providing critical economic justification for the updated screening guidelines.^5^ Together, these examples highlight the importance of integrating clinical evidence, cost-effectiveness, and policy to ensure that advances in GI translate into both improved patient outcomes and sustainable healthcare value.

## KEY CONCEPTS IN COST-EFFECTIVENESS ANALYSIS

CEA is a systematic approach to evaluating the relative costs and health outcomes of 2 or more interventions. The results are most often summarized as an incremental cost-effectiveness ratio (ICER), which represents the additional cost required to gain one unit of health benefit—commonly a quality-adjusted life year (QALY).^6^ Interpreting an ICER requires comparing it with a willingness-to-pay (WTP) threshold: If the ICER falls below the threshold, the intervention is considered cost-effective; if it exceeds it, the intervention is generally not.^7^ Importantly, WTP thresholds are context-specific and vary across healthcare systems and economic conditions.^8^ In the United States, thresholds are typically cited in the range of $50,000 to $200,000 per QALY, as reflected in the Institute for Clinical and Economic Review 2019 framework.^9^ To perform these analyses, structured approaches such as decision tree models, Markov models, and microsimulation are commonly used, and such analysis can be performed using platforms such as Excel, R, and TreeAge Pro.^10^ To strengthen the validity of such analyses, methods including deterministic sensitivity analyses and probabilistic sensitivity analyses are frequently used. These approaches allow investigators to test the robustness of results under a range of assumptions and account for uncertainty in clinical outcomes, costs, and model parameters—thereby providing more reliable evidence to inform clinical and policy decisions.

At the same time, it is important to recognize the limitations of CEA. These studies depend heavily on model assumptions, input data quality, and predefined WTP thresholds, all of which can vary across health systems and populations. Moreover, cost-effectiveness should never be interpreted in isolation; clinical judgment, patient preferences, and contextual factors must remain central to decision making. In practice, CEA is best viewed as one tool complementary to, but not a replacement for, clinical reasoning and individualized patient care.

Importantly, cost-effectiveness results are not universally transferable. The Institute for Clinical and Economic Review threshold for determining cost-effectiveness is highly context-specific and varies significantly by healthcare setting and country-level economic conditions. For instance, in the United Kingdom, the National Institute for Health and Care Excellence typically applies a threshold of £20,000–£30,000 per QALY, whereas in many low-income and middle-income countries, thresholds are often linked to gross domestic product per capita.^8,11^ These differences underscore the need to interpret CEA findings within the context of the healthcare system in which they will be applied.

## COST-EFFECTIVENESS IN CLINICAL PRACTICE

In a recent study evaluating noninvasive colorectal cancer screening strategies, Shaukat et al demonstrated that fecal immunochemical testing was the most cost-effective approach for reducing colorectal cancer incidence and mortality.^12^ However, when assuming a more realistic adherence rate of 60%, multitarget stool RNA testing emerged as more cost-effective than other molecular-based strategies. This analysis, conducted using a 10-year Markov model of disease progression, illustrates how CEA extends beyond simple cost-benefit comparisons, allowing for complex modeling that incorporates natural disease states, adherence patterns, and long-term outcomes.

Another example comes from the recent CEA by Hiramoto et al on the current treatment options for eosinophilic esophagitis.^13^ This study suggested that from the payer perspective, the 6-food elimination diet was the most effective and least costly first-line therapy whereas from the societal perspective, proton-pump inhibitors were more cost-effective. Payor perspective accounts for only direct medical costs such as medications, procedures, and hospitalizations, whereas societal perspective also considers indirect costs such as lost productivity or disability. This case highlights how the conclusions of cost-effectiveness analyses can differ depending on whether they are framed from the payer or societal perspective, underscoring the importance of context in interpreting results. Together, these examples demonstrate how CEA informs both preventive and therapeutic decision making in GI, providing a framework to balance patient outcomes with resource stewardship.

## THE FUTURE OF COST-EFFECTIVENESS IN GI

The role of cost-effectiveness will only grow in GI, particularly in advanced endoscopy. In recent years, randomized trials have increasingly compared procedural aspects of endoscopy, providing stronger evidence to inform both outcomes and economic analyses. With the rapid advent of artificial intelligence (AI) and precision medicine, there is significant scope to evaluate the cost-effectiveness of emerging interventions. For instance, AI-assisted endoscopy for polyp detection raises important questions about whether upfront costs are justified by long-term reductions in interval cancers, while the use of disposable vs reusable endoscopes underscores the trade-off between infection risk and cost. Future studies will need to address not only clinical performance but also whether these technologies provide value that justifies their widespread adoption.

Similarly, the emergence of novel microbiome-based therapeutics and precision medicine applications in inflammatory bowel disease introduces new opportunities and challenges for cost-effectiveness evaluation.^14,15^ Because these therapies often aim to improve functional outcomes such as patient quality of life alongside traditional clinical end points, such as endoscopic remission, incorporating robust quality-of-life assessments into cost-effectiveness analyses will be essential. Incorporating these patient-centered outcomes into an economic framework will help ensure that any additional costs are justified by meaningful and measurable benefits in patient quality of life.

However, translating cost-effectiveness evidence into policy is not without challenges, including misaligned reimbursement structures, variable payer incentives, and regulatory hurdles that may slow adoption of high-value innovations.^16^ The US Centers for Medicare & Medicaid Services took a notable step toward formalizing reimbursement pathways for AI in health care through the creation of an AI-specific Common Procedural Terminology code and the New Technology Add-On Payment for AI devices.^17–19^ While such developments in AI systems represent progress, they introduce new costs to the healthcare system, making rigorous cost-effectiveness evaluation of these technologies even more critical for widespread adoption.

For trainees, this evolution presents both a challenge and an opportunity. They will enter practice in a healthcare environment where decisions are judged by outcomes and costs, making early engagement with cost-effectiveness essential for preparing to deliver high-value care. Yet, discussions on value-based care and cost-effectiveness are often limited as a part of GI training. Incorporating these concepts into case discussions, journal clubs, and didactics can better prepare trainees to deliver high-quality, cost-conscious care. In conclusion, CEA is central to GI, shaping decisions from screening to advanced therapeutics. By incorporating economic evidence into clinical practice, gastroenterologists can ensure that patient care remains both high-quality and sustainable. As the field evolves, GI is well-positioned to lead by example in aligning innovation with value.

## DISCLOSURES

Author contributions: D.S. Dahiya is the article guarantor.

Financial disclosue: None to report.

Informed consent was obtained for this case report.

## ABBREVIATIONS:

AI: artificial intelligence
CEA: cost-effectiveness analysis
EMR: endoscopic mucosal resection
ESD: endoscopic submucosal dissection
GI: gastroenterology
ICER: incremental cost-effectiveness ratio
QALY: quality-adjusted life year
RNA: ribonucleic acid
US: United States
WTP: willingness-to-pay
